# Antimicrobial Susceptibility of Ceftazidime-Avibactam in Clinical Isolates of Carbapenemase-Producing Enterobacterales

**DOI:** 10.7759/cureus.92889

**Published:** 2025-09-21

**Authors:** Sarita Jain, Anamika Vyas, Parul Chaturvedi

**Affiliations:** 1 Microbiology, Geetanjali Medical College and Hospital, Udaipur, IND

**Keywords:** antimicrobial resistance, aztreonam synergy, carbapenemase-producing enterobacterales, ceftazidime-avibactam, metallo-β-lactamase

## Abstract

Background: Carbapenemase-producing Enterobacterales (CPE) are a major threat to public health around the world due to their extensive drug resistance mechanism. Ceftazidime-avibactam has emerged as a drug of choice for treating carbapenemase-producing organisms.

Objective: In this study, we aimed to evaluate the antimicrobial susceptibility of ceftazidime-avibactam against *Klebsiella pneumoniae* and *Escherichia coli* isolates that produce carbapenemases and to assess the synergistic effect of combining ceftazidime-avibactam with aztreonam against metallo-β-lactamase (MBL)-producing strains.

Methodology: It was a cross-sectional study, and data was collected over a period of six months in the Department of Microbiology, in a tertiary care teaching hospital of Southern Rajasthan. One hundred fifty clinical isolates, which were resistant to one of the carbapenems (imipenem, meropenem, or ertapenem) by the Kirby-Bauer disk diffusion method, were included in the study. All these isolates were subjected to carbapenemase detection by phenotypic methods, i.e., modified carbapenem inactivation method (mCIM) and ethylenediaminetetraacetic acid (EDTA)-carbapenem inactivation method (eCIM), and the results were analyzed to find out carbapenemase production as per Clinical and Laboratory Standards Institute (CLSI) 2023. Ceftazidime-avibactam susceptibility was determined using E-strips, and synergy with aztreonam was evaluated using disk approximation method.

Results: Among 150 isolates, 142 (94.7%) were confirmed as carbapenemase producers. Class A carbapenemases were detected in 89 (62.7%) isolates, class B MBLs in 38 (26.8%) isolates, and 15 (10.5%) showed mixed carbapenemase production. Ceftazidime-avibactam was effective against 87.6% of class A carbapenemase producers but only 15.8% against MBL producers. The combination of ceftazidime-avibactam and aztreonam showed synergistic activity in 84.2% of MBL-producing isolates.

Conclusion: Ceftazidime-avibactam shows excellent activity against class A carbapenemase-producing Enterobacterales but limited efficacy against MBL producers. The combination with aztreonam significantly enhances activity against MBL-producing strains which suggests a possible way to treat infections caused by these tough bacteria.

## Introduction

The misuse and overuse of antimicrobials have contributed significantly to the global rise of antimicrobial resistance (AMR), leading to the emergence of multidrug-resistant organisms, with carbapenemase-producing Enterobacterales (CPE) being among the most concerning pathogens [1]. The emergence and global dissemination of carbapenemase-producing *Klebsiella pneumoniae* and *Escherichia coli* have significantly compromised the effectiveness of carbapenems, which are considered last-resort antibiotics for treating serious infections caused by multidrug-resistant (MDR) Gram-negative bacteria [2].

Enterobacterales can resist the action of carbapenems through various mechanisms, the most common being the production of carbapenem-hydrolyzing enzymes (carbapenemases). These enzymes are classified into three main molecular classes based on the Ambler classification system. Class A carbapenemases, such as *Klebsiella pneumoniae* carbapenemase (KPC), are serine β-lactamases that can be inhibited by certain β-lactamase inhibitors. Class B metallo-β-lactamases (MBLs) require a zinc ion for their catalytic activity and include enzymes such as New Delhi metallo-β-lactamase (NDM), Verona integron-encoded metallo-β-lactamase (VIM), and imipenemase (IMP). Class D carbapenemases, primarily oxacillinase (OXA)-type enzymes, represent another significant group of carbapenem-hydrolyzing enzymes [3].

The clinical impact of CPE infections is significant, with studies reporting mortality rates ranging from 26% to 44% [4]. The limited treatment options available for treating CPE infections have mandated the development of new antimicrobial agents and combination therapies. The Food and Drug Administration (FDA) approved ceftazidime-avibactam in 2015, representing a significant advancement in the treatment of infections caused by carbapenemase-producing bacteria [5]. Ceftazidime-avibactam (CAZ-AVI), a combination of a third-generation cephalosporin and a beta-lactamase inhibitor, is one of the new antimicrobials that can be used against such carbapenemase-producing Gram-negative bacterial infections. Avibactam (AVI) is a non-beta-lactam beta-lactamase inhibitor with activity against class A and class C β-lactamases but has limited activity against metallo-β-lactamase (MBL) enzymes [6]. This limitation has led to investigations into combination therapy, particularly the age-old drug aztreonam with ceftazidime-avibactam, as it is stable against MBLs due to its potent affinity for penicillin-binding protein-3 (PBP3). However, its therapeutic benefit is impeded by the fact that MBL producers often secrete other class A, C, or D enzymes, against which it is ineffective, and these enzymes are inhibited by avibactam [7]. The objective of this study was to evaluate the antimicrobial susceptibility of ceftazidime-avibactam against *Klebsiella pneumoniae* and *Escherichia coli* isolates and to assess the synergistic effect of combining ceftazidime-avibactam with aztreonam against MBL-producing strains.

## Materials and methods

This cross-sectional study was carried out in the Department of Microbiology at a tertiary care teaching hospital of Udaipur, Rajasthan, for the period of six months after obtaining ethical clearance from the institutional ethical committee. A total of 150 non-duplicate clinical isolates of carbapenem-resistant Enterobacterales (CRE) were collected from various clinical specimens, including blood, urine, respiratory tract samples, and wound swabs. The isolates comprised *Klebsiella pneumoniae* (98 out of 150) and *Escherichia coli *(52 out of 150). These isolates were resistant to at least one of the carbapenems (imipenem, meropenem, or ertapenem) by the Kirby-Bauer disk diffusion method as per Clinical and Laboratory Standards Institute (CLSI) guidelines M100 33rd edition 2023.

Bacterial identification was confirmed using conventional biochemical methods and VITEK 2 system (bioMerieux, France). All isolates were stored at -80°C in tryptic soy broth with 15% glycerol until further analysis. Antimicrobial susceptibility testing was performed using the Kirby-Bauer disk diffusion method on Mueller-Hinton agar plates as per CLSI guidelines, M100 33rd edition, 2023 [8]. The following antibiotic disks were tested: imipenem (10 μg), meropenem (10 μg), ertapenem (10 μg), ceftazidime (30 μg), cefepime (30 μg), aztreonam (30 μg), amikacin (30 μg), gentamicin (10 μg), ciprofloxacin (5 μg), and trimethoprim-sulfamethoxazole (1.25/23.75 μg). *Escherichia coli* American Type Culture Collection (ATCC) 25922 and *K. pneumoniae* ATCC 700603 were used as quality control strains.

Modified carbapenem inactivation method in conjunction with the ethylenediaminetetraacetic acid (EDTA)-carbapenem inactivation method was performed on CRE isolates according to the CLSI guidelines to detect the presence of carbapenemase [8]. In brief, a 1-μL loopful of test bacterial inoculum was resuspended in a 2-mL tube of Tryptic Soy Broth (TSB). Another 1-μL loopful of test bacteria was resuspended in a 2-mL tube of TSB supplemented with EDTA at a final concentration of 5 mM (addition of 20 μL of 0.5M EDTA to 2 mL of TSB). A meropenem disk was placed in each tube, and the tubes were incubated at 35°C for 4 h. Subsequently, the disks were removed and applied to Mueller-Hinton (MH) agar plates, which were freshly plated with a 0.5 McFarland suspension of a carbapenem-susceptible *Escherichia coli* ATCC 25922 strain. Then the plates were incubated at 35°C for 16-20 h, and the mCIM and eCIM results were interpreted as described by CLSI23. The mCIM is considered negative if the zone size is ≥19 mm, positive if the zone size is 6-15 mm, or intermediate (defined as positive) if pinpoint colonies are present within a 16-18 mm zone. An isolate is positive for metallo-β-lactamase production when the eCIM zone size increases by ≥5 mm compared to the zone size observed for the mCIM and is considered negative for a metallo-β-lactamase if the increase in zone size is <4 mm.

Ceftazidime-avibactam susceptibility testing

Minimum inhibitory concentrations (MIC) of ceftazidime-avibactam were determined using E-test strips (bioMerieux, France) on Mueller-Hinton agar plates as per manufacturer’s instructions. Results were interpreted according to CLSI 2023 breakpoints: susceptible ≤8/4 μg/mL, intermediate 16/4 μg/mL, and resistant ≥32/4 μg/mL.

Aztreonam and ceftazidime-avibactam synergy testing

The combined action of aztreonam and ceftazidime-avibactam was evaluated using the disk approximation method. Aztreonam disks (30 μg) were placed at a center-to-center distance of 20 mm from ceftazidime-avibactam E-strips on Mueller-Hinton agar plates inoculated with test organisms. Enhancement of the aztreonam zone of inhibition towards the ceftazidime-avibactam strip indicated synergistic activity.

## Results

Out of 150 carbapenem-resistant Enterobacterales isolates, 98 (65.3%) were *K. pneumoniae* and 52 (34.7%) were *Escherichia coli*. In our study, the maximum isolates were obtained from urine (42%), followed by blood (28%), respiratory samples (18.7%), and wound swabs (11.3%) (Table 1).

**Table 1:** Distribution of bacterial isolates by specimen type Swabs include wound swab, vaginal swab, conjunctival swab, etc. Respiratory secretions include sputum, endotracheal secretions, bronchial aspirate, etc.

Specimen Type	Klebsiella​​ pneumoniae	Escherichia ​​​​​coli	Total (%)
Urine	38	25	63 (42.0)
Blood	28	14	42 (28.0)
Respiratory secretions	20	8	28 (18.7)
Swabs	12	5	17 (11.3)
Total	98	52	150 (100.0)

Phenotypic detection of carbapenemases

Among 150 isolates tested, 142 (94.7%) were confirmed as carbapenemase producers using the mCIM test. The eCIM test revealed that 38 (26.8%) isolates produce MBLs, while 89 (62.7%) isolates produce serine carbapenemases. Fifteen isolates (10.5%) showed characteristics suggesting mixed carbapenemase production (Table 2).

**Table 2:** Results of phenotypic carbapenemase detection mCIM: modified carbapenem inactivation method, MBL: metallo-β-lactamase.

Organism	mCIM Positive	Class A/C Carbapenemase	Class B (MBL)	Mixed Production
*Klebsiella pneumoniae* (n=98)	93 (94.9%)	58 (59.2%)	26 (26.5%)	9 (9.2%)
*Escherichia coli* (n=52)	49 (94.2%)	31 (59.6%)	12 (23.1%)	6 (11.5%)
Total (n=150)	142 (94.7%)	89 (62.7%)	38 (26.8%)	15 (10.5%)

Antimicrobial susceptibility pattern

Antimicrobial susceptibility testing revealed extensive resistance patterns among the carbapenemase-producing isolates. All isolates showed resistance to at least one carbapenem, with 89.3% being resistance to imipenem, 91.3% to meropenem, and 96.0% to ertapenem. High-level co-resistance were observed to other β-lactam antibiotics, with 92.0% showing resistance to ceftazidime and 88.7% to cefepime. Resistance rates to non-β-lactam antibiotics were also significant: gentamicin (78.7%), ciprofloxacin (84.0%), and trimethoprim-sulfamethoxazole (72.0%). Amikacin showed the lowest resistance rate at 45.3% (Table 3).

**Table 3:** Antimicrobial resistance patterns of carbapenemase-producing isolates TMP-SMX: trimethoprim-sulfamethoxazole.

Antibiotic	*Klebsiella pneumoniae* (%)	*Escherichia coli* (%)	Overall (%)
Imipenem	88.8	90.4	89.3
Meropenem	92.9	88.5	91.3
Ertapenem	97.0	94.2	96.0
Ceftazidime	94.9	86.5	92.0
Cefepime	91.8	82.7	88.7
Aztreonam	89.8	84.6	88.0
Amikacin	48.0	40.4	45.3
Gentamicin	80.6	75.0	78.7
Ciprofloxacin	86.7	78.8	84.0
TMP-SMX	75.5	65.4	72.0

Ceftazidime-avibactam susceptibility

**Figure 1:**
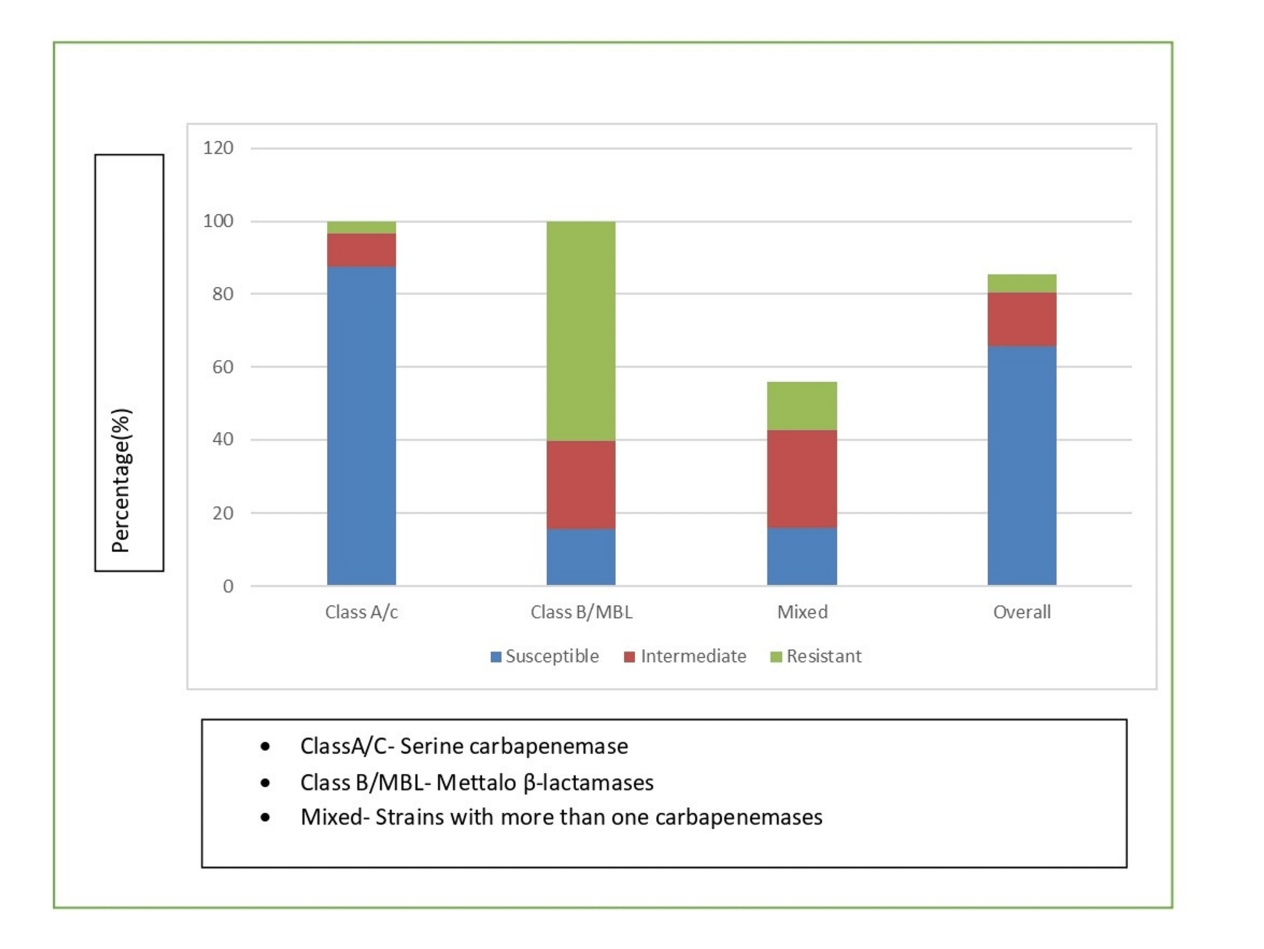
Ceftazidime-avibactam susceptibility by carbapenemase type E-test methodology performed according to manufacturer's instructions (bioMérieux, France). Interpretive criteria based on Clinical and Laboratory Standards Institute (CLSI) 2023 guidelines: susceptible ≤8/4 μg/mL, intermediate 16/4 μg/mL, and resistant ≥32/4 μg/mL.

Ceftazidime-avibactam demonstrated variable susceptibility depending on the carbapenemase type. Among the 89 isolates producing class A/C carbapenemase, 78 (87.6%) were susceptible to ceftazidime-avibactam, eight (9.0%) showed intermediate susceptibility, and three (3.4%) were resistant. In contrast, among the 38 MBL-producing isolates, only six (15.8%) were susceptible, nine (23.7%) showed intermediate susceptibility, and 23 (60.5%) were resistant. The MIC50 and MIC90 values for class A/C carbapenemase producers were 2/4 μg/mL and 8/4 μg/mL, respectively (Figure 1).

Aztreonam-ceftazidime-avibactam synergy

**Figure 2:**
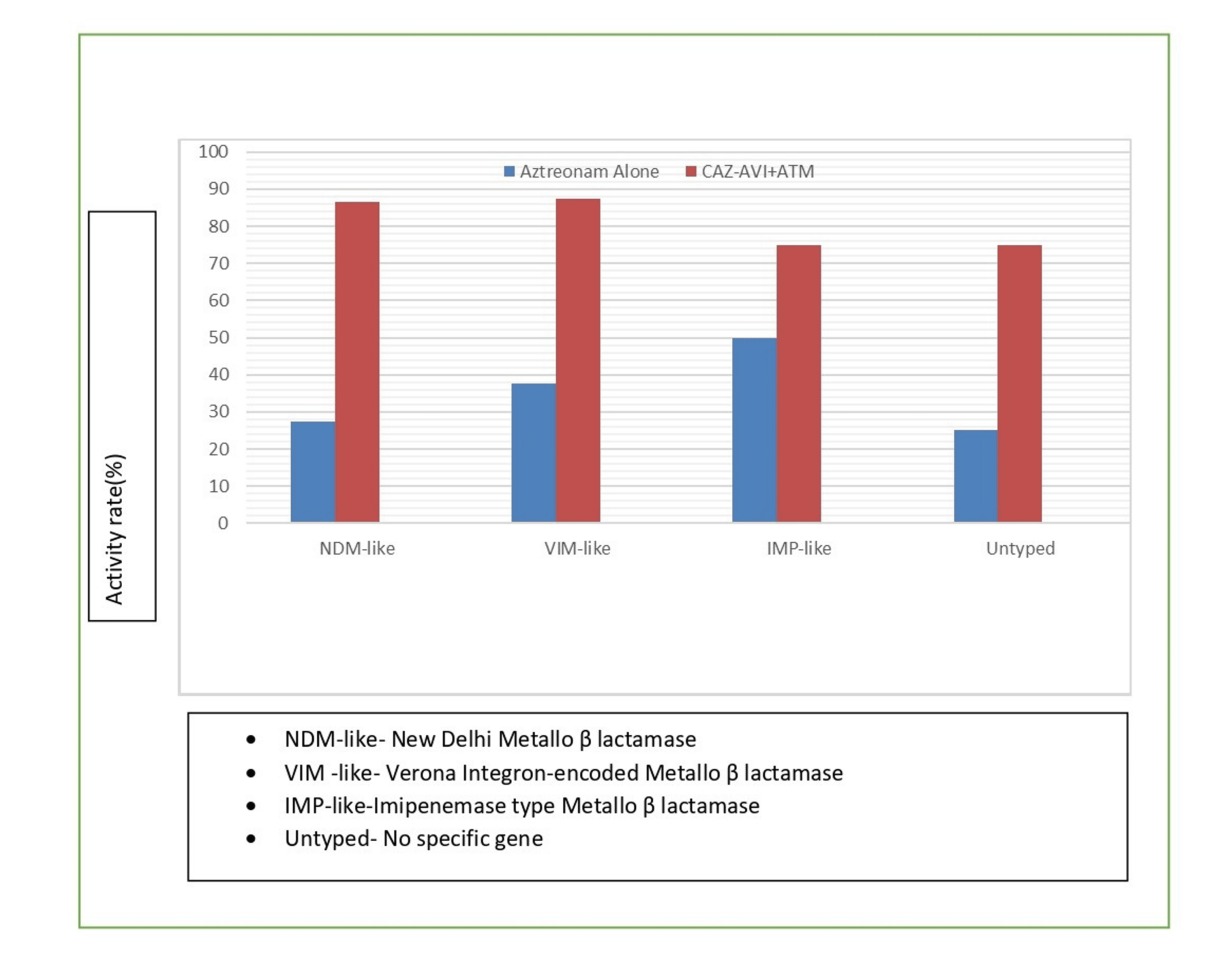
Synergy of ceftazidime-avibactam plus aztreonam against MBL-producing isolates Disk approximation method performed according to standardized protocol. Aztreonam disks (30 μg) placed 20 mm center-to-center from ceftazidime-avibactam E-test strips. Synergy defined as enhancement of aztreonam zone of inhibition toward the ceftazidime-avibactam strip. MBL typing based on phenotypic characteristics using eCIM methodology. MBL: metallo β-lactamase, eCIM: EDTA-carbapenem inactivation method, CAZ-AVI+ATM: ceftazidime-avibactam plus aztreonam.

The combination of aztreonam with ceftazidime-avibactam demonstrated significant synergistic activity against MBL-producing isolates; 32 (84.2%) showed synergistic activity when aztreonam was combined with ceftazidime-avibactam, compared to only 12 (31.6%) that were susceptible to aztreonam alone. The synergy was particularly clear in isolates with high ceftazidime-avibactam MICs (>16/4 μg/mL), where 28 (87.5%) out of 32 isolates showed synergistic enhancement (Figure 2).

## Discussion

This study provides comprehensive insights into the antimicrobial susceptibility patterns of ceftazidime-avibactam against carbapenemase-producing Enterobacterales, with particular emphasis on the differential activity against various carbapenemase types. The high prevalence of carbapenemase production (94.7%) among our carbapenem-resistant isolates aligns with previous studies that have reported carbapenemase-mediated resistance in Enterobacterales [9].

The predominance of class A/C carbapenemases (62.7%) in our study is consistent with the global epidemiology of carbapenemase-producing Enterobacterales, where KPC-type enzymes remain prevalent in many regions [10]. However, the significant proportion of MBL producers (26.8%) reflects the increasing global dissemination of NDM-type enzymes, particularly in clinical settings where broad-spectrum antibiotic use is common [11].

The excellent activity of ceftazidime-avibactam against class A/C carbapenemase producers (87.6% susceptibility) confirms the therapeutic potential of this agent for treating infections caused by KPC-producing organisms. These findings are consistent with clinical studies that have demonstrated the efficacy of ceftazidime-avibactam in treating serious infections caused by carbapenemase-producing bacteria [12]. The MIC50 and MIC90 values of 2/4 μg/mL and 8/4 μg/mL, respectively, for class A/C producers indicate that the most isolates remain well within the susceptible range, suggesting that standard dosing regimens would be appropriate for most infections.

Conversely, the limited activity of ceftazidime-avibactam against MBL producers (15.8% susceptibility) was expected, given that avibactam does not inhibit metallo-β-lactamases. This finding emphasizes the critical importance of rapid carbapenemase detection in clinical laboratories to guide appropriate antimicrobial therapy [13]. The most significant finding of our study is the demonstration of synergistic activity between ceftazidime-avibactam and aztreonam against MBL-producing isolates. The 84.2% synergy rate observed in our study provides strong evidence for the clinical utility of this combination. The mechanism underlying this synergy is well established: while aztreonam remains stable against MBLs, it is hydrolyzed by extended-spectrum β-lactamases (ESBLs) and AmpC β-lactamases that are commonly co-produced by MBL-positive isolates. The addition of avibactam inhibits these enzymes, thereby restoring aztreonam activity [14]. The differential synergy rates observed among different MBL types (NDM-like: 86.4%, VIM-like: 87.5%, and IMP-like: 75.0%) may reflect variations in the co-resistance mechanisms present in these isolates. The slightly lower synergy rate in IMP-producing isolates could be attributed to additional resistance mechanisms or variations in β-lactamase expression levels [15].

The clinical implications of our findings are substantial. For infections caused by class A/C carbapenemase producers, ceftazidime-avibactam monotherapy represents an excellent therapeutic option with a high likelihood of clinical success. However, for infections caused by MBL producers, combination therapy with aztreonam should be strongly considered. The implementation of rapid diagnostic methods for carbapenemase detection becomes crucial in guiding these treatment decisions [16].

Several limitations should be acknowledged in our study. First, the phenotypic methods used for carbapenemase detection while following CLSI guidelines may not provide the specificity of molecular methods for precise enzyme identification. Second, the study design does not provide information about clinical outcomes, which would be valuable for validating the therapeutic implications of our findings.

The emergence of resistance to ceftazidime-avibactam, particularly in class A carbapenemase producers, has been reported and represents a growing concern [17]. The 3.4% resistance rate observed in our class A/C producers, while low, warrants continuous monitoring and emphasizes the importance of antimicrobial stewardship programs to preserve the efficacy of this valuable agent. Future research directions should include molecular characterization of carbapenemase genes to provide more precise epidemiological data, evaluation of the pharmacokinetics and pharmacodynamics of ceftazidime-avibactam and aztreonam combinations, and clinical outcome studies to validate the therapeutic efficacy of these approaches. Additionally, investigation of emerging resistance mechanisms and the development of rapid diagnostic methods for carbapenemase typing will be crucial for optimizing patient care.

## Conclusions

This study demonstrates that ceftazidime-avibactam exhibits excellent antimicrobial activity against carbapenemase-producing Enterobacterales with class A/C carbapenemases, with 87.6% of isolates showing susceptibility. However, its efficacy against MBL-producing isolates is severely limited, with only 15.8% susceptibility. The combination of ceftazidime-avibactam with aztreonam shows remarkable synergistic activity against MBL producers, with 84.2% of isolates demonstrating enhanced susceptibility.

These findings have important clinical implications for the management of infections caused by carbapenemase-producing Enterobacterales. The rapid identification of carbapenemases is essential for guiding appropriate antimicrobial therapy. While ceftazidime-avibactam monotherapy is highly effective against class A/C carbapenemase producers, combination therapy with aztreonam should be considered for MBL-producing isolates.

The study reinforces the critical need for continued surveillance of antimicrobial resistance patterns, implementation of robust infection control measures, and development of novel therapeutic strategies to combat the growing threat of carbapenemase-producing bacteria. Healthcare institutions should prioritize the establishment of rapid diagnostic capabilities for carbapenemase detection and to optimize patient outcomes and preserve the efficacy of available antimicrobial agents.
